# Mr. Charles Bell's Consultations and Cases Illustrative of His Papers on the Nerves

**Published:** 1827-04

**Authors:** 


*Appendix to the Papers on the Nerves, republished from the Philosophical Transactions.* By CHARLES BELL; containing Consultations and Cases Illustrative of the Facts announced in those Papers. 8vo, pp. 140, with a Plate of the Respiratory Nerves. Longman and Co. 1827.

THE discoveries of Mr. Bell, in respect to the nervous system, are now universally known to the profession, and their application to practice has been often made of late, by others as well as by the ingenious author himself. The present little volume is constituted chiefly by examples of this application of certain highly interesting views of the nervous system, (peculiar to Mr. Bell) at the bed-side of sickness. Its contents will therefore be eagerly perused by the author's medical brethren, and will, we are sure, contribute to their improvement. With the hope of widening the circle of perusal, we shall here endeavour to condense into as small a space as possible, an analysis of some of the more important of these "CONSULTATIONS AND CASES."

*Case* 1. Paralysis about the face is always alarming to the patient, and very often to the practitioner, unless the latter be acquainted with the discoveries of Mr. Bell, when he will soon discriminate between partial paralysis and that dependent on affection of the encephalon. The following case was, and we believe is, under the care of Dr. Gregory. The patient was a sailor, 45 years of age, who had been out of health for five years previously. Three years ago he was seized with paralysis of the lower extremities, after a few days illness. He soon recovered and pursued his business. After the paraplegia, he was subject to ague for eighteen months. Had had an abscess in his right ear, which burst and discharged matter, for a considerable time. In August, 1826, a substance started from his ear, while in the act of coughing; and three weeks afterwards was observed to have his face drawn to one side, and his medical man warned him that he was going to have another attack of paralysis, and advised him to be cupped, blistered, &c. These measures do not appear to have had any effect, and the following were the phenomena presented, when he came under Dr. Gregory.

"All the muscles of the right side of his face, which are controlled by the influence of the portio dura, or respiratory nerve of the face, are completely paralysed. He cannot elevate his eyebrow nor frown; there is a line nearly in the centre of his forehead, dividing the bulging of the muscles on the left side from the smooth uncontracted state of those on the right side. He cannot close the eyelids of the right eye; they remain always open: when he makes the attempt to close them, we see the eyeball rolling upwards. The secretion of tears is very abundant, so as to render this eye more glistening than the other: he complains of the inconvenience produced by its continually weeping; he also attributes a dimness of vision in the right eye to this cause. From the nature of his occupations, he is constantly troubled by the dust getting into this eye; but he has acquired a readiness of pulling down the eyelid with his finger, to defend it. His daughter says, that when she has seen him asleep, only the white of his eye was visible.

"His right nostril is collapsed. The muscles of the cheek and mouth are relaxed and dragged to the left side. When he speaks, the cheek flaps like a blind before an open window, and if he attempt to utter a word with peculiar emphasis, the air escapes from the corner of his mouth, like the whiff of a person smoking. He sometimes experiences a difficulty of swallowing, at the moment when the morsel is thrown back into the fauces.

"The sensibility of the right side of his face is natural. When he clenches his jaws, the masseter muscles can be felt equally hard and contracted on both sides of his face. He can protrude his tongue, and twist it to either side. He is deaf in the right ear." 11.

The peculiarity of the foregoing case consists in the occurrence of paralysis twice in the same individual, though it is evident that they were of different kinds, and from different causes. The facial paralysis must be considered as arising from affection of the portio dura in its course through the temporal bone.

*Case* 2. Mr. Bell was consulted (by letter) respecting a man, 50 years of age, who, for 20 years, had been affected with an involuntary contraction of the muscles on one side of his face, drawing up the angle of the mouth, and giving to the palpebræ a winking motion, so remarkable as to be seen at a considerable distance. The man was determined to have some nerves cut to prevent such a disagreeable state. Except once, he never had any pain during these convulsive actions; but then it was so severe as to resemble tic douloureux. The consultant (a medical man) proposed a division of the nerves, if Mr. Bell approved of the operation. Another gentleman came to town to have the branches of the fifth pair, on one side of the face, divided, in order to balance the paralytic features. "A singular consequence would have resulted from such an operation. The patient would have been deprived of sensibility on one side of his face, and of motion on the other."

"If the subject of the present consultation had submitted to have the nerve cut, his eye would have remained open, for the *attollens palpebræ* being supplied by the third nerve, and the *orbicularis palpebrarum* by the seventh, the cutting of the latter would have paralysed the eyelids: they would have remained open, and the eye would have become inflamed, and probably opaque. There would have been greater deformity, and blindness to boot." 15.

Mr. Bell wrote, of course, to the medical consultant in Scotland, warning him of the effects of dividing the portio dura, which operation would only paralyze certain muscles, without affecting the sensibility of the face, which is dependent on the fifth pair of nerves. Mr. B. recommended medical rather than surgical treatment.

" 'You will find this twitching to depend a good deal on the state of the digestion. Anodyne liniments rubbed in the course of the nerve, and pressure to limit the motion of the parts spasmodically affected, I have found attended with advantage. The pressure, which restrains this spasmodic motion, tends to break that habit, on which, in a great measure, it at length depends, however originally produced: and indeed it is this circumstance, the continuance of the symptoms for twenty years, which forbids me being sanguine in the expectation of your effecting a cure.' " 18.

*Case* 3. This was detailed by Dr. Fergusson, of Baker Street. The patient was a sailor, aged 35 years, who had been ill with scrofulous affections for three years. Two years previously to the date of statement, he became deaf of his left ear, and had subsequently a discharge from it. Abscesses afterwards formed in various parts of the body, and one betwixt the mastoid process and the angle of the jaw, in the left side. In a short time after the abscess, the left side of the face became paralytic, and the eye-lids (of that side) stood open, nor could they be closed by any voluntary effort of the patient, except by aid of the hand. He could chew equally well on both sides, and the sensation of touch remained equally acute in all parts of the face. When he attempted to close his eyes, the left eye-ball was turned up.

It is evident that the portio dura was here involved in the seat of the abscess, and that it had partaken of the inflammation, thus causing the paralysis.

*Case* 4. The particulars of the following case were transmitted to our author by Mr. Jackson, of Sheffield. Mary Unwin, aged 22, in her second pregnancy, has complained, for some time, of spasms in the lower extremities, constipation of the bowels, and slight dulness over the eyes.

" On examining the countenance, a singular distortion of the features is most apparent. The mouth is drawn to the right side, and the nose evidently inclines in the same direction. She was asked to put the forehead in action as in frowning, and then was presented the appearance of wrinkles across the right side of the forehead, whilst the opposite side was even and perfectly unmoved. In sleep, the right eyelids are closed as usual, but the left eye remains uncovered. She appears to have no power over the muscles, whose office it is to move the eyelids of the left side.

" 'There is little (I think no) difference in the sensibility of the two sides of the face. There is occasionally a dimness of vision of the left side, owing probably to the circumstance of the globe of the eye not being lubricated with the tears, as is the case with the opposite one.

"'The patient states that she experiences pain on the left side of her neck, and at the root of the ear of that side; but there is no swelling nor marked evidence of inflammation existing in these parts. On pressing on the branch of the *portio dura*, or, as you have termed it, the respiratory nerve of the face, especially in the situation of the parotid gland, no uneasiness is experienced. The iris moves in obedience to the stimuli of light, and the tongue possesses its natural movement. In fact, there is no paralysis in any part of the body, excepting in those parts specified above, and which are supplied with nervous influence by the *portio dura*.'" 29.

Mr. Jackson having taken the proper view of the case, confined the treatment to moderate evacuations, instead of resorting to excessive depletion, as would formerly have been done, when the brain would have been considered in danger.

*Case* 4. We give the particulars of this case, because the man is about the Middlesex Hospital, and may be seen at any time.

It was casually observed, when this man brought his wife to the hospital for a paralytic stroke, that the left side of his face was much distorted, with great wasting of the muscles.

"'He was examined by Mr. Bell, before the pupils of the hospital, and it proved to be a case of paralysis of the portio dura. The two sides of his forehead presented a very striking contrast: the right side was furrowed with deep wrinkles, which were more strongly marked when he frowned: a large fold of the skin was prolonged down upon the same side of his nose, which marked the descending slip of the occipito frontalis muscle; the left side of his forehead was perfectly smooth, the skin appearing to be stretched tightly over the bone, and there was no motion of the integuments in the act of knitting or elevating his eyebrows. His eyelids were quite motionless. When he was desired to wink, this eye remained open, and the cornea was elevated so as to be quite hid under the upper eyelid. This eye appeared a little duller than the other, yet he says he never had any disease in it. He cannot see so clearly with it as with the other eye. The left nostril is collapsed, and has not that fulness which the right possesses. Some power of acting with his cheeks seems to remain, as in whistling there is a slight quivering observed. Although his lips are dragged to the right side, yet they do not appear to be totally deprived of muscular power: he can grasp pretty firmly the point of the little finger, when it is introduced into the left angle of his mouth. The muscles of the neck are perfect: the fibres of the platysma myoides start out when he puts it into action. The skin is naturally sensible. He states that he has had this affection since he was a child. He has had no deafness; nor any disease which he can remember to have preceded this distortion of his countenance.'" 30.

Passing over several cases, partly Mr. Bell's own, and partly taken from Descot's dissertation on local affections of nerves, we come to a train of cases, where the nerves of the trunk, neck, and throat, especially those belonging to the respiratory system, were affected. We shall glance at a few of them.

*Case* 5. An unmarried lady, nearly 70 years of age, had enjoyed uninterrupted good health up to the present illness, with the exception of occasional, and short attacks of gout in the feet and knees. A few months ago she had some difficulty in moving the tongue, and in expressing some particular words, which difficulty has increased, so that now she has lost her speech altogether, from inability to move her tongue. The organ looks soft and pulpy, but retains its sense of taste and of feeling. The deglutition is impaired, and she is occasionally distressed with a sense of suffocation, in attempting to swallow food. The features of the face are natural, and the skin sensible. The saliva occasionally flows from her mouth.

This was the case submitted to Mr. Bell, by Dr. Robinson, of Preston. This case is descriptive, Mr. B. observes, of a paralytic affection of the 9th nerve, a cerebral and motor nerve, and therefore more alarming, as proceeding from the brain, and threatening apoplexy. It is evident in this case that the fifth pair of nerves is unaffected, as the taste and feeling of the tongue remain entire. Mr. Bell recommended nauseating medicines—leeches under the mastoid process—a seton across the neck—and any local appearance of gout to be encouraged.

We knew an officer, then about 40 years of age, who frequently lost completely the power of uttering a single word, for hours together, though every other faculty and function was in a state of integrity. The first attack came on while in the middle of his dinner, and alarmed him exceedingly. He retired immediately into another room, and sent for the writer of this article. He wrote down on a piece of paper that he was quite speechless. He had little or no command of motion in the tongue, and as he was of very plethoric habit, we certainly apprehended impending apoplexy. We bled him largely, but this made no difference in the paralysis. When his arm was tied up, he fainted, and, on recovering, turned sick, and vomited up some most intensely acid matters. The moment his stomach was cleared, the power of speech returned. After this, he had many attacks of a similar kind, and they always proved to be connected with acidity in the stomach, with which complaint he was more distressed than any individual we ever attended. He was obliged to be always taking magnesia or alkalies. He retired from the service on account of his health, and he is now alive, after 18 years retirement. But we lately heard that he had an attack of apoplexy, last year, and is now completely hemiplegiac.

*Case* 6. A boy, 10 years of age, had been subject from childhood to a pain in his ear. This, about a year ago, became so intense in his left ear, that he had no rest, night or day. The pain extended to his head and face, affecting the forehead, and the sockets of the teeth. After this his left eye became affected, and he lost his sight. He recovered by large bleedings, leeches, and blisters. There has often been much discharge from the ear, attended with fever, great pain, and sometimes phrenzy. In one of these attacks, he had a strong convulsive fit, and on recovering sense, he was found to be speechless. He was admitted into the Middlesex on the 7th of October. About a week afterwards, he threw himself down in a violent passion, and from that moment he was entirely deaf. Since that period, his left arm has become useless. He can move his fingers, but not his arm. Part of the member is acutely painful when touched.

" 'He is now brought under Mr. Bell's care, who has made a particular examination of his condition. The actions of respiration are perfect. When he smiles, there is no inequality in the action of the muscles of the face. He is reported to make noise enough in laughing. When cupped, he hollowed out, and they thought every moment he would speak, yet there was no articulate sound. The boy is acute, and understands every thing communicated to him by writing. When by this means he is asked to speak, and when the throat is grasped during the effort, there is not the slightest motion perceptible in the muscles of the tongue. Yet he can masticate and swallow with ease; he can nearly touch the point of his nose with his tongue; he can turn it down to the chin and sideways. When his surgeon's name is written, and he is asked to pronounce it, he remains fixed with his mouth open. When by signs he is told to close his lips in the manner necessary to pronounce the letters *b* and *p*, and when he is then asked to pronounce these letters, there seems an utter inability. The consent of action between the chest, larynx, and mouth, seems to be lost.' " 92.

The patient was repeatedly purged with calomel and jalap—had leeches behind the ears—fomentations and blisters to the sides of the head. His condition, as to health, and the power of the arm, was much bettered, but he continued mute and deaf, and afterwards left the hospital, towards the end of December. He was brought to Mr. Bell's house in the July following, when his mother informed Mr. B. that three months previously he had suddenly recovered both speech and hearing, after a discharge of matter from his mouth, which appeared to come from his head. He had been nine months dumb. His sense of hearing was now morbidly acute.

"This case of Hill's is not demonstrative, for happily there was no dissection to ascertain the precise nature of the injury to the nerves; but it is illustrative. There appears to have been an abscess, originally produced by disease of the temporal bone, and affecting the nerves of the base of the brain, first affecting the fifth nerve, and then spreading its influence to the seventh and ninth. If the disease had produced its influence mechanically and by pressure, there would have been no obscurity, and one side only would have been affected; but I imagine the inflammation had disturbed the operations of the nerves, without altogether destroying their influence, deranging, for instance, the fine associations necessary to speech, without arresting the action of the muscles of the tongue. It is remarkable, that the bursting out of matter, probably from the Eustachian tube, had such an instantaneous and simultaneous effect in restoring both hearing and speech." 96.

*Cases 7 & 8.* Mr. Bell alludes to some curious cases of young females, affected with distressing symptoms about the throat, which are extremely puzzling. He was consulted in the case of a young lady, who had "a convulsive barking noise like a cough, excepting that the larynx was alone affected, and there was no conforming action in the pharynx, velum, and lips. She would sometimes cough naturally in the intervals of this noise; but this natural coughing did not interrupt the return of the unpleasant hard bark, at the rate of ten times in the minute. It ceased when she was asleep, but the moment she was awake, the family heard the noise, intolerable from repetition. It continued a month, and returned three successive winters." We recorded a remarkable case of this kind, some time ago, which was seen by many physicians of this metropolis, one of whom bespoke a sight of the dissection, as he was confident there was fatal ulceration of the larynx! The young lady is now alive and well. Dr. Sims, Dr. Armstrong, Sir Astley Cooper, and many others, will remember this case.

In one instance of this kind, the spasm of the glottis was so continued and severe that the attendants called on Mr. Bell to perform laryngotomy!

"All the subjects of these odd cases, which we do not understand, get well. This is consolatory to a patient, certainly, but not very satisfactory to ourselves. Ought it not to be a question, what nervous affections are consequent on trivial irritation? Without entering on the question, whether deranged health be followed by the imperfect and deranged action of the uterine system, or whether the latter be the primary disorder—*the ovaria are the source of irritation;* and the consequences are exhibited through the most susceptible system of nerves, the respiratory system. Hence the disorder of stomach, the spasms, globus, the difficulty of deglutition, the aphonia: hence the affection of the countenance, the tears, the sobbing, and spasms of the eyes and face, and throat, and chest, and stomach." 99.

We are rather at a loss to know on what foundation Mr. Bell grounds the positive assertion, that "*the ovaria are the source of irritation*" in these cases. We have little doubt, indeed, that the uterine system is generally the seat of the original irritation; but why the ovaria should be selected as the exclusive seat, is not quite so apparent.

*Case* 9. Mr. Bell visited a lady who lay on an inclined plane, totally incapable of moving; yet her breathing was easy, and her countenance animated, although she could not speak. "Here was a case of the paraplegia of the older authors—a palsy affecting the whole body below the neck." As the patient lived and breathed, under these circumstances, it is clear that the respiratory muscles were not affected—and, consequently, that they are supplied with a distinct set of nerves.

*Case* 10. Anne Roper was admitted into the Middlesex, in June, 1825, for a strange spasmodic affection, which, before the discoveries of Mr. Bell—and, perhaps, even at the present moment, would puzzle a conjuror. It is necessary to say that her bowels were out of order, and her catamenia irregular.

"'The condition of this woman is very peculiar: inspiration is performed with a sudden spasmodic action, in her common breathing: she is also affected at intervals with more violent spasms, and respiration is then hurried. On the commencement of a paroxysm, she bends her body slightly forwards, and thus prepares herself for the attack: her nostrils are widely dilated, the angles of her mouth are dragged forcibly downwards, there is a constriction of the throat, the shoulders and chest rise convulsively, as when a person has cold water poured upon the head; there is a deep and violent inspiration, attended with a sniffing of the nostrils, from the air being inhaled through them only, and not through the mouth. The fibres of the platysma myoides start into view, there is a quick rising and falling of the pomum Adami, the sterno-cleido-mastoideus, and trapezius on both sides, act powerfully in fixing the head and elevating the shoulders.

"'The spasmodic action of these muscles exists to a considerable degree constantly, yet it increases in paroxysms which last for a few minutes so severe that she is deprived of the power of speech, and seems to be almost suffocated. These paroxysms recur at irregular intervals.

It was observed by the attendants, that when she was excited by walking about the ward or by replying to our questions, they returned more frequently.

"'She could move her head with perfect freedom when we requested her, but still the spasmodic action continued. She also raised either shoulder, or twisted her face to one side, when she was desired. This woman continued under the care of the physician for about a month, and was discharged cured.'" 106.

No other explanation or history of this case is given, than that she was treated successfully by Dr. Southey. The means are not stated; but there can be no doubt that the indications of cure hinged on the improvement of the digéstive and uterine functions.

Mr. Bell's little volume closes with some "additional observations to the cases of injury of the spine," of which we shall give the particulars of the principal case.

*Case* 11. James Palmer, aged 16, was admitted into the Middlesex on the 4th October, 1825, suffering, as was supposed, from a blow on the head. It turned out, however, that this injury was a mere slap of the hand on the top of the head, without any violence. The patient stated that, three weeks previously, he had caught a violent cold, with stiffness and swelling round the neck. When this subsided, there came on a swelling at the back of the neck, which continued to increase, until he felt a numbness in parts of his arms and fingers, and also in his leg. The use of the right arm and leg was, at length, lost; and he was then brought to the hospital, exhibiting the following phenomena, viz.—a swelling round the spine, on the back of the neck, apparently a thickening of the ligaments and cellular membrane around the bones. This tumefaction is greatest on the right side—complains of no pain, and can bend his neck to a certain degree; but requires assistance to move either arm or leg of the right side; the left is less affected. *8th Oct.* An issue was made on each side of the cervical spine, by incisions, which bled freely. Next visit he was better, and could move his arm and leg. This amelioration soon disappeared. Leeches were ordered, under the idea, that the temporary relief had been owing to the loss of blood. *14th.* He is worse—complains of more numbness, and can move neither leg nor arm—when he attempts to move his head, he has great pain, increased when the head is permitted to fall forward. Means were taken to give as much rest as possible to the inflamed vertebræ, and leeches, issues, purgatives, &c. were attended to. *18th.* The boy breathes rather better, and has more power of motion in the left leg. Leeches, &c. continued. *19th.* Not so well. Lies a little twisted, and breathes laboriously. *20th.* Worse. *25th.* Breathing difficult—complains of a sense of weight on the chest—voice more feeble. The sterno-cleido-mastoideus muscle appears in strong action—abdominal muscles totally inactive and loose. *Jan. 7th.* The extenuation of the abdominal muscles permits the peristaltic motion of the intestines to be seen through them. He is sensible of pressure on the stomach, but not so on the intestines. He can give no impulse to the expiration of air, but expires merely by the falling of the chest. In sleep his thighs are involuntarily drawn up, and he has no power of pushing them down.

*September* 1st. No note has been taken of this lad's case for some months; but the improvement is remarkable. The motion of the right arm first returned, and then that of the left. He then began to move his legs, first the right, and next the left. At last he managed to get out of bed, and crawl about the ward. He is now able to go on crutches. No other treatment was pursued than attention to the bowels, to prevent constipation; and keeping up active issues in the neck, with rest.

"This case of Palmer is very interesting, and abundantly confirms the result of the cases of fracture of the spine. By the progress of inflammation beginning in the vertebræ, and propagated to the spinal marrow, we see the function of the spinal marrow slowly debilitated, and at length the symptoms coming to resemble those produced by the crushing of the spinal marrow by the broken vertebræ. But here we can observe the gradual failure of strength, and the consequence of inactivity, in the wasting of the muscles. The most remarkable effect of this, was the possibility of seeing the intestines moving, and the relaxed abdominal muscles partially rising and falling according to the distention and contraction of the intestinal canal. This state of the abdomen permitted us to examine the stomach, and to ascertain that its sensibility was entire; and it is fair to conclude, through the influence of the par vagum. The branches of this nerve to the stomach, like its subdivisions to the larynx and pharynx, are in possession of two functions; the peculiar sensibility of the part is bestowed, and the arrangement of the muscles is formed, through its influence. It must be remembered, however, that this double office is from its receiving additional branches from the spinal nerves just as it is emerging from the base of the skull. From the first instance given in this section, where a lady was insensible below the neck, it may be presumed that the influence of the lungs, and heart, and stomach, on the respiratory system of muscles, exciting to the act of breathing, was propagated through the par vagum.

"As the symmetrical system of nerves to the trunk became impaired, the muscles supplied with the accessory respiratory nerves became more excited, and rose higher into action. At the same time, the voice became feeble. This is easily understood, for the strength of the voice results from the impetus with which the breath is expelled. In this case, the active muscular power of expelling the breath was lost, with the other voluntary powers of the trunk and extremities; and by this we see the importance to life of these accessory nerves of respiration, for they continuing to possess power over the diaphragm, serratus magnus anticus, trapezius, and sterno-cleido mastoideus, they supplied a force of inspiration sufficient to preserve life, until amendment took place in the spinal marrow, and common spinal nerves. No one, I apprehend, will be bold enough to affirm, that if the muscles of the neck and trunk had been as entirely deprived of action as the abdominal muscles were in this case, the patient could have survived by the mere action of the diaphragm." 135.

A very good plate of the respiratory nerves and muscles accompanies the volume.

These cases and consultations of Mr. Bell will be read with considerable interest by the profession—not only by the inquisitive physiologist, but by the plodding practitioner. The only drawback upon the consultations, is one inseparable from such documents—namely, our ignorance, generally speaking, of the final issue of each case. Some of these deficiencies, we think, Mr. Bell might have supplied—and he certainly might have been more communicative, in several instances, respecting the treatment employed; as, for example, in the case of Ann Roper, under his care and that of Dr. Southey. Mr. Bell, however, was more intent on elucidating the physiology and pathology of the superadded system of nerves, than on any therapeutical measures employed in removing morbid affections. We are grateful for what he has laid before the public, and hope he will persevere, through good and evil report, in the path of professional reputation which he has so long trodden, with honour to himself, and advantage to the public. We shall here close this article with the concluding passage in Mr. Bell's volume.

"And now, in parting with my reader, I congratulate him that we have a profession that furnishes us continually with new objects of interest, and makes us independent of all encouragement, whether from men of science purely, or from that part of the public that follow science from fashion. They are occupied with matters which they conceive to be of superior consequence: we feel and know that we are engaged in pursuits, that are both higher as matters of science, and infinitely more important to humanity." 140.

